# Lipocalin 2 links inflammation and ankylosis in the clinical overlap of inflammatory bowel disease (IBD) and ankylosing spondylitis (AS)

**DOI:** 10.1186/s13075-020-02149-4

**Published:** 2020-03-18

**Authors:** Aifeng Lin, Robert D. Inman, Catherine J. Streutker, Zhenbo Zhang, Kenneth P. H. Pritzker, Hing Wo Tsui, Florence W. L. Tsui

**Affiliations:** 1 Institute of Medical Science, University of Toronto, Toronto, Ontario Canada; 2 Krembil Research Institute, University Health Network, Toronto, Ontario Canada; 3 KeyIntel Medical Inc, Toronto, Ontario Canada; 4 Department of Immunology and Department of Medicine, University of Toronto, Toronto, Ontario Canada; 5 Department of Laboratory Medicine and Pathobiology, University of Toronto, Toronto, Ontario Canada; 6 Li Ka Shing Institute, St. Michael’s Hospital, Toronto, Ontario Canada

**Keywords:** Ankylosing spondylitis, Inflammatory bowel diseases, Lipocalin 2, PPARγ, *ank/ank* mice

## Abstract

**Background:**

Little is known about the mechanisms underlying the clinical overlap between gut inflammation and joint ankylosis, as exemplified by the concurrence of inflammatory bowel diseases (IBD) and ankylosing spondylitis (AS). As dysbiosis may serve as a common contributor, the anti-microbial pleiotropic factor lipocalin 2 could be a potential mediator due to its roles in inflammation and bone homeostasis.

**Methods:**

Baseline colonic pathology was conducted in the *ank/ank* mouse model. Serum lipocalin 2 was analyzed by ELISA, in *ank/ank* mutants versus C3FeB6-A/A^w-j^*wt/wt*, in patients with concurrent AS-IBD, AS alone, IBD alone, or mechanical back pain, and in healthy controls. In the *ank/ank* mouse model, the expression of nuclear receptor peroxisome proliferator-activated receptor gamma (PPARγ) was examined by real-time PCR. Intraperitoneal injection was done with the PPARγ agonist rosiglitazone or antagonist bisphenol A diglycidyl ether for four consecutive days. Serum levels of lipocalin 2 were examined on the sixth day.

**Results:**

This study showed that the *ank/ank* mice with fully fused spines had concurrent colonic inflammation. By first using the *ank/ank* mouse model with progressive ankylosis and subclinical colonic inflammation, confirmed in patients with concurrent AS and IBD, elevated circulating lipocalin 2 levels were associated with the coexisting ankylosis and gut inflammation. The intracellular pathway of lipocalin 2 was further investigated with the *ank/ank* mouse model involving PPARγ. Colonic expression of PPARγ was negatively associated with the degree of gut inflammation. The PPARγ agonist rosiglitazone treatment significantly upregulated the serum levels of lipocalin 2, suggesting a potential regulatory role of PPARγ in the aberrant expression of lipocalin 2.

**Conclusions:**

In summary, lipocalin 2 modulated by PPARγ could be a potential pathway involved in concurrent inflammation and ankylosis in AS and IBD.

## Background

The clinical overlaps between gut inflammation and joint ankylosis, such as those found in inflammatory bowel disease (IBD) and ankylosing spondylitis (AS), have long been recognized. For example, AS and IBD are linked, as up to one third of IBD patients develop articular disease with features of AS [1], while approximately 10% of AS patients have coexisting clinical IBD [2]. In addition, there is asymptomatic subclinical gut inflammation in 40–60% of patients with AS, both macroscopically and microscopically [3, 4]. Also, 44% of IBD patients fulfill the criteria for inflammatory joint disease [5].

Although attempts have been made to unravel the mechanisms underlying the concurrence of gut-joint manifestations, the pathogenesis remains unclear. Disturbance of gut microbiome may serve as a common potential environmental trigger. Recently, dysregulation of gut bacteria (dysbiosis), which is a recognized feature of IBD [6], has been discovered in AS patients and in a rat model of AS [7, 8]. Inoculation with microbiota from IBD patients triggered more severe colitis in germ-free interleukin 10-deficient mice than those treated with intestinal content from healthy controls [9]. Furthermore, 20% of patients with reactive arthritis who were triggered by gastrointestinal infections by *Campylobacter* spp., *Yersinia* spp., *Salmonella* spp., and *Shigella* spp. eventually progressed to AS [10].

Acting as an anti-microbial acute phase reactant, the levels of lipocalin 2 (mouse Lcn2/human LCN2) are increased to limit the growth of pathogens upon infection [11]. Lipocalin 2 could be one of the mediators in the gut-joint axis, owing to its pleiotropic properties in inflammation and bone remodeling [12, 13]. It is produced by multiple cell types in different tissues, including the gut and joint [14, 15]. Serum LCN2 has been reported to be elevated and associated with disease activity in patients with IBD [16]. Moreover, the relationship of murine Lcn2 and peroxisome proliferator-activated receptor gamma (PPARγ) in colitis models and lipid studies reveals a relevant intracellular pathway of aberrant Lcn2 expression regulated by PPARγ [17–22]. PPARγ is a key nuclear receptor regulating both gut inflammation and mesenchymal stem cell differentiation into osteoblasts [23, 24]. The activation of PPARγ can be modulated by agonists such as rosiglitazone (Rosi) and antagonists such as bisphenol A diglycidyl ether (BADGE) [25, 26].

In this study, we aimed to decipher whether lipocalin 2 and its association with PPARγ mediate the gut-joint axis, by using the *ank/ank* mutant mouse model with concurrent progressive ankylosis and subclinical gut inflammation. Our study showed that elevated serum Lcn2 in *ank/ank* mice was associated with coexisting gut inflammation and spinal ankylosis. Previously, *ank/ank* mutant mouse model led us to discover a distinctive serological feature in patients with AS [27]. In our study, patients with concurrent AS and IBD confirmed the findings in *ank/ank* mice, indicating that LCN2 might serve as a link of the gut-joint axis in AS and IBD. The role of PPARγ in modulating Lcn2 expression in the *ank/ank* mouse model suggests a potential intracellular molecular pathway in the gut-joint linkage, which could shed light on potential therapeutic targets for patients with concurrent gut-joint manifestations.

## Methods

### Animals and study design

Heterozygous mice on a background of C3FeB6-A/A^w-j^ (*ank+/−*) were used for breeding to obtain *ank/ank* mice and *wt/wt* littermates. *ank/ank* mice and C3FeB6-A/A^w-j^*wt/wt* littermates were cohoused until 4–5 weeks of age. To eliminate variation in the progression of ankylosis, studies were undertaken in animals at an age > 16 weeks when all had fused peripheral and axial joints.

For baseline studies of the *ank/ank* mouse model, 4–5-month-old *ank/ank* mice (*n* = 40; 25 males vs 15 females) and age-matched C3FeB6-A/A^w-j^*wt/wt* littermates (*n* = 21; 10 males vs. 11 females) were used. Whole length colon tissues were collected from *ank/ank* mice and C3FeB6-A/A^w-j^*wt/wt* littermates. Blood samples were collected and allowed to clot at 4 °C. Samples were then centrifuged for 10 min at 3000*g*, and serum samples were stored at − 70 °C until use. Fecal samples were prepared following an established protocol with modifications [28]. Briefly, 20–50 mg of fecal sample from each mouse was homogenized in PBS containing 0.1% (v/v) Tween20 (10 mg fecal sample/100ul PBST) by vortexing for 20 min at room temperature. Mixtures were then centrifuged for 10 min at 12,000 rpm at 4 °C. Supernatant was collected and stored at − 20 °C until analysis.

For PPARγ manipulation studies in the *ank/ank* mouse model, 44 *ank/ank* mice (19 males vs 25 females) and 54 C3FeB6-A/A^w-j^*wt/wt* littermates (30 males vs 24 females) were used. BADGE or Rosi (Sigma Aldrich Company) was dissolved in 5% DMSO. Mice were randomized into three treatment groups: (1) 5% DMSO, (2) 30 mg/kg BADGE, and (3) 10 mg/kg Rosi. Intraperitoneal administration of BADGE, Rosi, or DMSO was conducted daily for 4 days. Mice were sacrificed on the 6th day. Serum samples were collected and stored at − 70 °C.

All animals were housed in the specific pathogen-free animal facility at the Krembil Research Institute according to the guidelines of Canadian Council of Animal Care.

### Human patients

AS patients who met the modified New York classification criteria for the disease [29] (with at least unilateral sacroiliitis scores of 3 or 4) and mechanical back pain (MBP) patients were recruited during 2004 to 2016 from the Toronto Western Hospital AS clinic. MBP patients had no clinical evidence of inflammatory back pain and no radiographic evidence of sacroiliitis. There are 462 patients with AS alone and 57 AS patients with clinical IBD. One hundred fifty-eight healthy controls (HC) were also recruited concurrently at the Toronto Western Hospital. Serum samples of 85 patients with IBD but not AS were a gift by Dr. Mark Silverberg from the Mount Sinai Hospital. The latest Modified Stoke AS Scoring System (mSASSS) of each patient was available. Demographic features of different cohorts are summarized in Table 1.

**Table 1:** Demographic features of different cohorts

	AS alone	AS-IBD	MBP	IBD	HC
*N* (male/female)	462 (350/112)	57 (44/13)	52 (26/26)	85 (85/0)	158 (106/52)
HLA-B27 positivity	78% (357/456)	56% (32/57)	0% (0/52)	N/A	0% (0/158)
CRP (mg/L)	13.9 ± 21.1	15.4 ± 17.3	2.13 ± 2.8	N/A	N/A
ESR (mm/hour)	13.5 ± 15.6	14.4 ± 17.2	6.2 ± 4.9	N/A	N/A
BASDAI	4.8 ± 2.5	4.5 ± 2.6	4.8 ± 2	N/A	N/A
Biologics*	43% (200/462)	74% (42/57)	N/A	N/A	N/A
Disease duration (years)	2–62	2–53	N/A	N/A	N/A

*Biologics include infliximab, adalimumab, golimumab, and etanercept

### Staining and scoring of baseline gut pathology

Gut tissues were cut open, washed with PBS, and rolled up (*Swiss roll* technique) to evaluate the gut from the proximal to the distal end. Tissues were then fixed in 4% formalin overnight and processed for hematoxylin and eosin staining.

Pathological scoring was done following an established system with modifications [30]. Briefly, an experienced pathologist blinded to the experimental conditions scored tissues according to the following: (1) acute inflammation (0, none; 1, rare diffuse or small focal clusters; 2, increased numbers of neutrophils, easily identified; 3, severe inflammation, usually associated with erosions or ulcerations), (2) chronic inflammation (0, none; 1, focal and or minimally increased lymphocytes/plasma cells, particularly in the deep lamina propria; 2, moderate inflammation; 3, severe chronic inflammation, usually with loss of crypts, foreshortened crypts, or enough chronic inflammation to push crypts apart), and (3) the percentage of bowel involved (0, none; 1, < 20%; 2, 20–50%; 3, > 50%). The primary pathology score is the sum of all three scores above (0–9).

To further validate the primary scoring system, a secondary scoring system was established based on published studies [31, 32]. Briefly, tissues were scored according to the following: (1) location of inflammation (0, none; 1, mucosal; 2, transmural), (2) the degree of hyperplasia in colon (0, none; 1, 1–50% increase in height; 2, 51–100% increase in height; 3, > 100% increase), (3) mucin depletion (0, none; 1, mild loss of goblet cells; 2, moderate loss; 3, severe loss), (4) granulomas (0, absent; 1, present), (5) number of foci (0, none; 1, < 5; 2, 5–10; 3, > 10), and (6) architecture distortion (0, none; 1, rare foci of arch changes (< 5%); 2, moderate with 5–50% glands showing changes; 3, > 50% of glands showing changes). The secondary pathology score is the sum from scores of the six categories above (0–15). Semi-quantification of neutrophils and plasma cells was calculated on the average of cell counts per field from ten random fields per mouse.

### Quantification of serum and fecal Lcn2 levels by ELISA

Serum samples from each mouse at baseline and following treatment were thawed and analyzed at the same time to minimize inter-assay variation. Lcn2 levels were measured by an ELISA kit according to manufacturer’s protocol (R&D). Supernatants from fecal samples were diluted 50-fold, while serum samples were diluted 100-fold using kit-recommended reagent diluent (1% BSA in PBS). Plates were read at 450 nm with a correction at 570 nm. Total protein of supernatant extracted from fecal samples was measured using the Pierce Coomassie Plus Protein Assay with a BSA standard. Fecal Lcn2 levels were further compared with the total protein to control for water content variable in the wet feces.

### Baseline colonic PPARγ expression analysis

Total RNA was isolated from colons (whole thickness) using TRIzol reagent (Invitrogen) according to the manufacturer’s protocol. The extracted RNA was purified with the RNeasy Mini Kit (Qiagen), which included an on-column DNase I treatment step, according to the manufacturer’s protocol. RNA purity was determined by spectrophotometry (A260/A280 and A260/A230). RNA was considered intact on a denaturing gel with sharp 28S and 18S rRNA bands and the 28S rRNA bands twice as intense as the 18S rRNA bands. cDNA was synthesized from 2μg of total RNA using the High-Capacity cDNA Reverse Transcription Kit (Applied Biosystems) according to the manufacturer’s recommendation. Relative quantification of gene expression was then performed by SYBR green real-time PCR. Primers specific for PPARγ and reference gene GAPDH are as follows: PPARγ forward 5′ ACGTTCTGACAGGACTGTGTG 3′; PPARγ reverse 5′ TGATGTCAAAGGAATGCGAGTG 3′; GAPDH forward 5′ TGTGTCCGTCGTGGATCT 3′; GAPDH reverse 5′ CCTGCTTCACCACCTTCTTGA 3′. SYBR green real-time PCR was performed in 384-well plates with a reaction volume of 10 μl using the ABI PRISM 7900HT Fast System (Applied Biosystems) and the standard cycling conditions, as per the manufacturer’s instructions. Each gene was run in duplicate. Data was normalized to the reference gene GAPDH and expressed as a fold change versus C3FeB6-A/A^w-j^*wt/wt* with a pathology score 8 (the highest score found in our study), using the 2^−ΔΔCt^ method.

### Statistics

Student’s *t* test, one-way analysis of variance (ANOVA) followed by Bonferroni’s multiple comparison tests, and Pearson’s correlation coefficient tests were carried out using GraphPad Prism 5 program. A *p* value of less than 0.05 was considered significant. Data are presented as mean ± standard error.

## Results

### Coexisting colonic inflammation and ankylosis in *ank/ank* mice

Homozygous *ank/ank* mice are normal at birth. Ankylosis is first observed in distal digits at 4–5 weeks of age. Progressive ankylosis develops from peripheral to axial skeleton, with a rigid spine evident in the homozygous mice by 12–16 weeks of age. To maintain uniform degree of ankylosis, 4–5-month-old *ank/ank* mice with fully established spinal ankylosis were used, with age-matched C3FeB6-A/A^w-j^*wt/wt* littermates as controls.

**Fig. 1:**
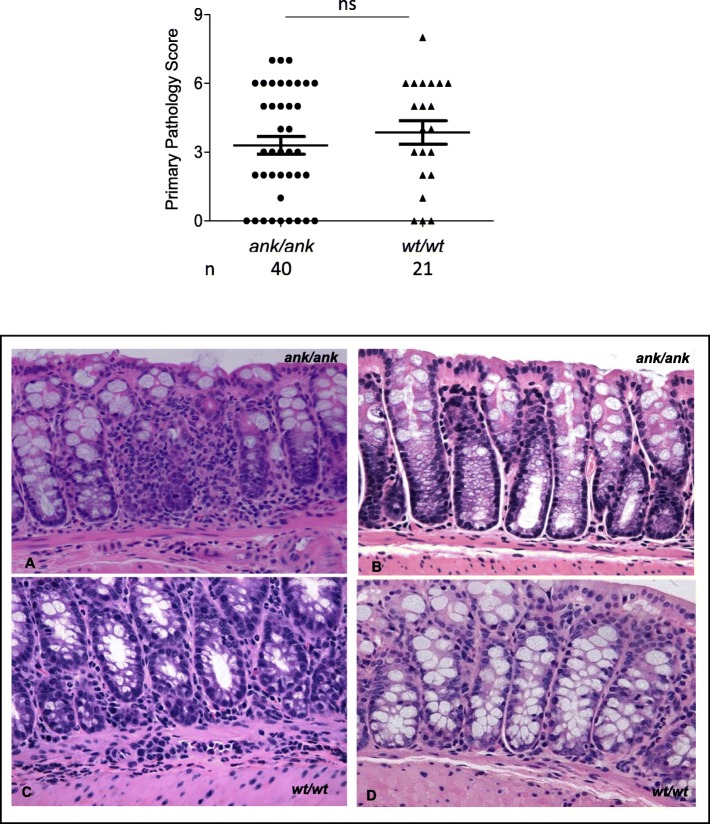
Subclinical colonic inflammation in *ank/ank* mice and C3FeB6-A/A^w-j^*wt/wt* littermates at baseline. *Top panel*: Total pathology score (0–9) of 4–5-month-old *ank/ank* mice (*n* = 40) and C3FeB6-A/A^w-j^*wt/wt* controls (*n* = 21). Student’s *t* test was used. *Bottom panel*: **a** Representative histopathological features of colons (× 200) (done by hematoxylin and eosin staining) from a *ank/ank* mouse with patchy colon inflammation (score 7). **b***ank/ank* mouse with no colon inflammation (score 0). **c** C3FeB6-A/A^w-j^*wt/wt* mouse with a small focus of mucosal chronic inflammation (score 5), and **d** C3FeB6-A/A^w-j^*wt/wt* mouse with normal colon (score 0)

Various degrees of subclinical inflammation were observed in both *ank/ank* and C3FeB6-A/A^w-j^*wt/wt* mice (Fig. 1 top panel). The primary pathology score was the aggregate of the acute inflammation score, the chronic inflammation score, and the percentage of bowel involved on a 0–9 scale. There were comparable scores in *ank/ank* (3.3 ± 1.6; interquartile range (IQR), 1.75–6) vs. C3FeB6-A/A^w-j^*wt/wt* littermates (3.9 ± 1.5; IQR, 2–6). None of the mice had clinically significant gut inflammation as defined by diarrhea.

A representative pathology section (with a score of 7) from an *ank/ank* mouse reveals a large number of inflammatory infiltrates in the crypt area with significant structural change in the colon (Fig. 1 bottom panel a). A representative section (with a score of 5) from a C3FeB6-A/A^w-j^*wt/wt* mouse differs from the *ank/ank* mouse in that the infiltrates are primarily located at the lamina propria area with a small focus of mucosal chronic inflammation (Fig. 1 bottom panel c). Gut tissues with no inflammation (with scores of 0) from *ank/ank* and C3FeB6-A/A^w-j^*wt/wt* mice are included for comparison (Fig. 1 bottom panel b and d).

In light of the different locations of infiltrates discovered in the gut of *ank/ank* and C3FeB6-A/A^w-j^*wt/wt* mice, further analysis with a more detailed secondary scoring system addressed whether there were pathological differences between *ank/ank* mice and C3FeB6-A/A^w-j^*wt/wt* littermates. There was a significant (*p* < 0.01) difference in the degree of mucin depletion between *ank/ank* mice (0.03 ± 0.03) and C3FeB6-A/A^w-j^*wt/wt* controls (0.3 ± 0.1) (Suppl. Table 1). There was no difference between the two groups of mice in the severity of hyperplasia, number of foci, and architecture distortion, as well as depth of inflammation. No granulomas were detected in any of the mice. There were comparable neutrophil and plasma cell counts in both groups (Suppl. Fig. 1).

The primary scoring system was further validated with the secondary scoring system. There was a significant correlation of the primary vs secondary pathology scores (*r*^2^ = 0.8, *p* < 0.0001) (Suppl. Fig. 2A). Furthermore, neutrophils (Suppl. Fig. 2B) and plasma cell (Suppl. Fig. 2C) counts were also positively correlated with the primary pathology scores (*r*^2^ = 0.2, *p* = 0.0003 and *r*^2^ = 0.4, *p* < 0.0001, respectively). The scores mentioned in the following text refer to the primary pathology scores if not specified.

### Baseline serum Lcn2 levels are elevated in mice with coexisting ankylosis and severe subclinical colonic inflammation

**Fig. 2:**
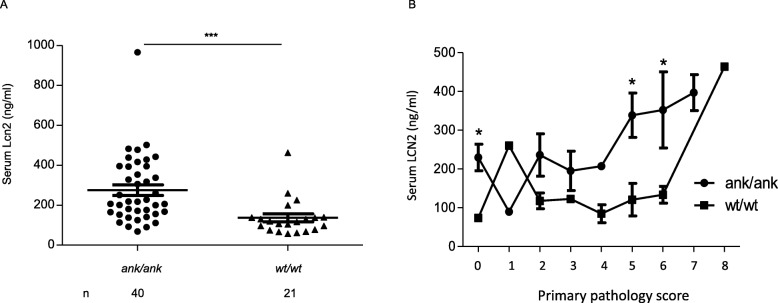
Serum Lcn2 levels in mice with various degrees of colon pathology and with or without ankylosis. **a** Detection of serum Lcn2 levels in 4–5-month-old *ank/ank* (*n* = 40) and C3FeB6-A/A^w-j^*wt/wt* (*n* = 21) mice. Student’s *t* test was used. **b** Comparison of serum levels of Lcn2 in *ank/ank* vs. C3FeB6-A/A^w-j^*wt/wt* mice with different pathology scores. Student’s *t* test was used (* represents significant difference of *ank/ank* vs. C3FeB6-A/A^w-j^*wt/wt* mice with the same pathology scores)

Serum Lcn2 analysis in animals revealed significantly (*p* < 0.001) higher Lcn2 levels in *ank/ank* mice (276 ± 27 ng/ml) than in C3FeB6-A/A^w-j^*wt/wt* animals (137 ± 20 ng/ml) (Fig. 2a). Mice with the same pathology scores were further compared (Fig. 2b).

For lower pathology scores (score 2–4), Lcn2 levels were comparable in *ank/ank* mice vs. C3FeB6-A/A^w-j^*wt/wt* littermates with the same score. However, for animals with pathology score 5, *ank/ank* mice had significantly higher Lcn2 levels (339 ± 57 ng/ml, *p* = 0.03) compared to C3FeB6-A/A^w-j^*wt/wt* controls (121 ± 42 ng/ml). Notably, similar findings were observed for mice with score 6. Lcn2 levels in *ank/ank* mice were positively correlated with the pathology scores (*r*^2^ = 0.13, *p* = 0.02). But no significant correlation was found between the C3FeB6-A/A^w-j^*wt/wt* littermates and the degree of gut inflammation. Thus, serum Lcn2 levels were distinctly elevated only when higher degrees of gut inflammation coexisted with spinal ankylosis, as was present only in the *ank/ank* mice. Subclinical colonic inflammation alone in *wt/wt* mice might not be sufficient to trigger an elevation of serum Lcn2. However, there was no association of fecal Lcn2 levels and the severity of gut involvement in neither *ank/ank* mice (*r*^2^ = 0.04, *p* = 0.26 and *r*^2^ = 0.0001, *p* = 0.9) nor C3FeB6-A/A^w-j^*wt/wt* littermates (*r*^2^ = 0.1, *p* = 0.16 and *r*^2^ = 0.25, *p* = 0.06), before and after controlling for the total protein amount (Suppl. Fig. 3).

### Serum levels of LCN2 are elevated and associated with concurrent IBD and AS in patients

**Fig. 3:**
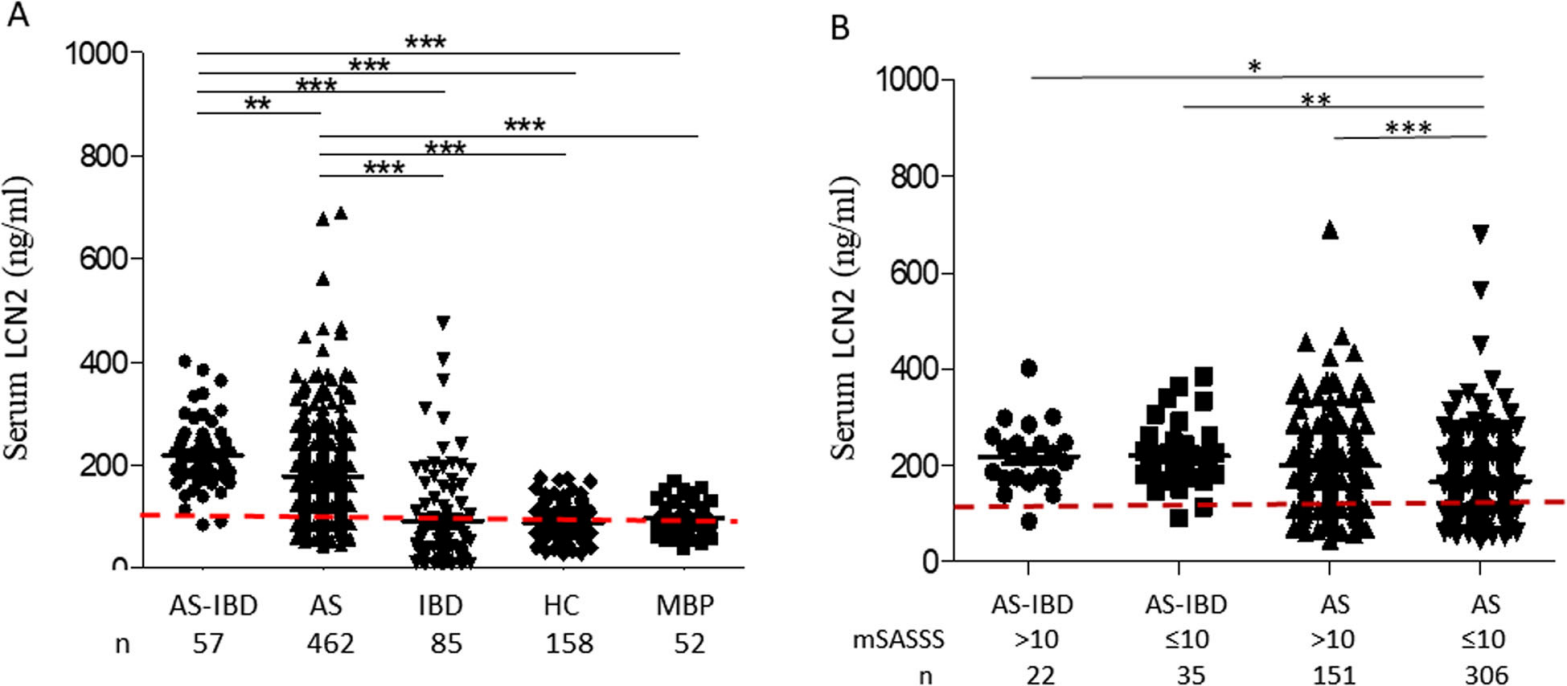
Serum LCN2 levels in human patients. **a** Detection of serum LCN2 levels in patients with AS-IBD (coexisting AS and IBD; *n* = 57), AS (ankylosing spondylitis; *n* = 462), IBD (inflammatory bowel disease; *n* = 85), HC (healthy control; *n* = 158), and MBP (mechanical back pain; *n* = 52). **b** Detection of serum LCN2 levels of different degrees of ankylosis (mSASSS > 10 vs. MSASSS ≤ 10), in AS patients with or without IBD. One-way analysis of variance (ANOVA) followed by Bonferroni’s multiple comparison test was used

To investigate the relationship of LCN2 with ankylosis and gut involvement in patients, LCN2 levels were measured in patients with AS-IBD, AS alone, and IBD alone and compared to healthy controls (HC) and mechanical back pain (MBP) patients. Patients with coexisting AS and IBD had significantly higher levels of serum LCN2 (220 ± 9 ng/ml) compared to those with AS alone (178 ± 4 ng/ml; *p* < 0.001) and IBD alone (91 ± 10 ng/ml; *p* < 0.0001), respectively. AS patients also had significantly higher serum levels of LCN2 than IBD (*p* < 0.0001), HC (88 ± 2 ng/ml; *p* < 0.0001), and MBP patients (97 ± 4 ng/ml; *p* < 0.0001) (Fig. 3a).

The availability of the mSASSS in the patients enabled us to analyze whether an association of LCN2 and severity of spinal ankylosis existed. The degree of ankylosis was defined as more severe ankylosis (mSASSS > 10) and less severe ankylosis (mSASSS ≤ 10), in patients with AS-alone and AS-IBD. Figure 3b showed that higher LCN2 levels (199 ± 8 ng/ml; *p* < 0.001) is associated with more severe ankylosis in patients with AS alone, suggesting a relationship between ankylosis and circulating LCN2. Results from correlation analysis of mSASSS and LCN2 levels in AS alone patients is consistent with this finding (*r*^2^ = 0.04, *p <* 0.0001) (Suppl. Fig. 4). Moreover, in patients with less severe ankylosis, higher LCN2 levels are detected in those with coexistent IBD (220 ± 11 ng/ml; *p* < 0.01), confirming the contribution of gut inflammation in the elevation of LCN2.

### Baseline colonic PPARγ expression is negatively related with the degree of colonic inflammation in mice

**Fig. 4:**
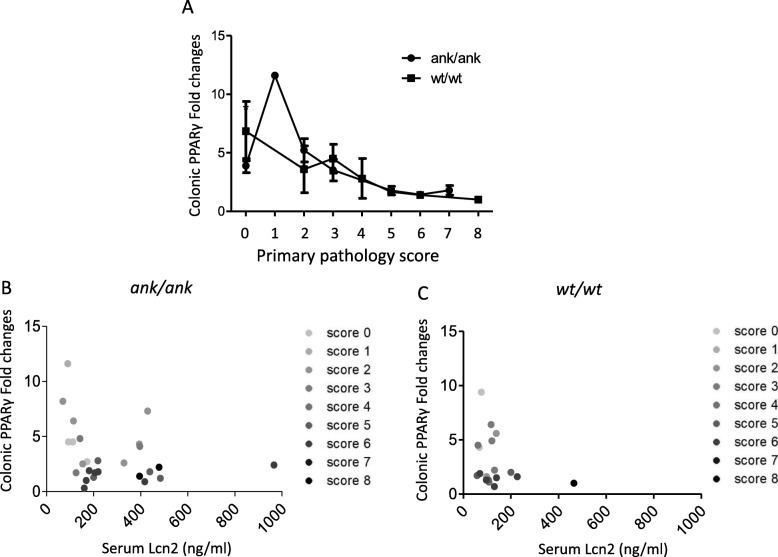
Baseline expression of colonic PPARγ and the association with serum Lcn2 in mice. A. Comparison of the expression of PPARγ in *ank/ank* and C3FeB6-A/A^w-j^*wt/wt* mice with different pathology scores. Student’s *t* test was used. B. The association of colonic PPARγ expression and serum Lcn2 versus gut pathology in *ank/ank* mice. C. The correlation of colonic PPARγ expression and serum Lcn2 versus gut pathology in C3FeB6-A/A^w-j^*wt/wt* mice

To investigate whether there is an association between PPARγ and Lcn2, the expression of PPARγ at baseline was determined in the colons of mice with different degrees of gut involvement (Fig. 4a). In both *ank/ank* mice and C3FeB6-A/A^w-j^*wt/wt* animals, the expression of colonic PPARγ had a negative correlation with the pathology scores (*r*^2^ = 0.41, *p =* 0.0004 and *r*^2^ = 0.81, *p =* 0.0005, respectively). However, there was no difference in the expression of PPARγ in *ank/ank* vs. C3FeB6-A/A^w-j^*wt/wt* animals, with any same pathology scores.

The expression of colonic PPARγ was further associated with serum Lcn2 levels in *ank/ank* (Fig. 4b) and C3FeB6-A/A^w-j^*wt/wt* mice (Fig. 4c). As the association of pathology scores with serum Lcn2 is opposite to that with colonic PPARγ expression, animals with different pathology scores were color-coded, with higher scores being darker colors in the graphs. When comparing animals with different degrees of gut inflammation within the same genotype, animals with higher pathology scores appeared to have lower PPARγ expression but higher Lcn2 levels than those with lower scores.

### Lcn2 was upregulated in response to a PPARγ agonist in mice

**Fig. 5:**
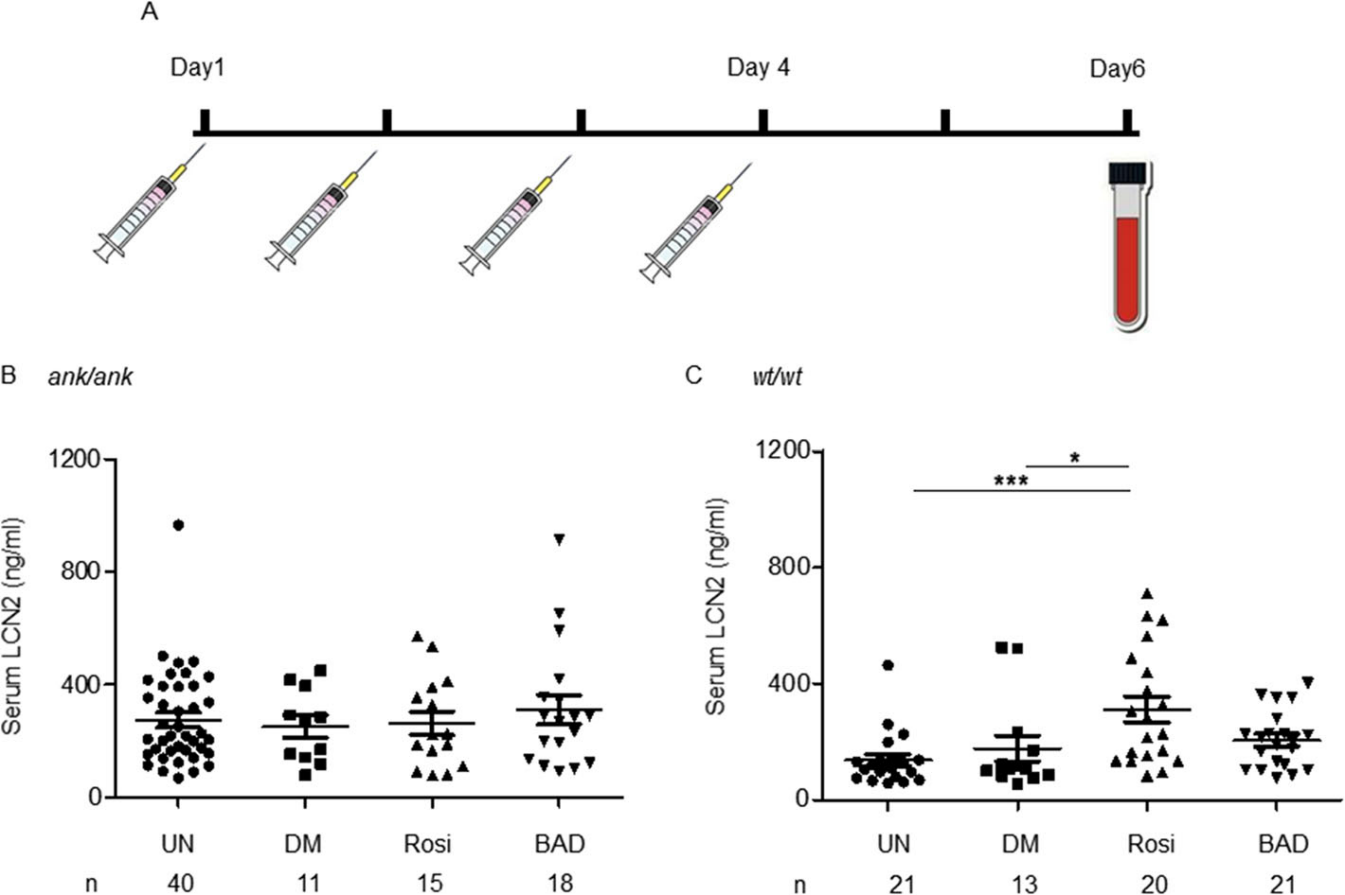
Serum Lcn2 levels after rosiglitazone (Rosi) or bisphenol A diglycidyl ether (BADGE) treatments in mice. A. Study design. B. Serum Lcn2 levels of *ank/ank* mice before and after Rosi/BADGE treatments. C. Serum Lcn2 levels of C3FeB6-A/A^w-j^*wt/wt* mice before and after Rosi/BADGE treatments. UN: untreated animals (baseline); DM: 5% DMSO control animals; Rosi: 10 mg/kg body weight rosiglitazone treated animals; BAD: 30 mg/kg body weight bisphenol A diglycidyl ether (BADGE) treated animals. One-way analysis of variance (ANOVA) followed by Bonferroni’s multiple comparison test was used

To detect whether PPARγ plays a role in Lcn2 expression, intraperitoneal injections with either a PPARγ agonist (Rosi) or an antagonist (BADGE) were performed in both *ank/ank* and C3FeB6-A/A^w-j^*wt/wt* animals for 4 consecutive days (Fig. 5a). Animals were sacrificed on the sixth day. Serum levels of Lcn2 were analyzed. No DMSO (a carrier of Rosi/BADGE) effect on Lcn2 was detected in either *ank/ank* or C3FeB6-A/A^w-j^*wt/wt* mice. Neither Rosi nor BADGE treatment changed the levels of Lcn2 in *ank/ank* mutants (Fig. 5b). In C3FeB6-A/A^w-j^*wt/wt* mice, Rosi treatment significantly augmented the levels of serum Lcn2 (313 ± 44 ng/ml) compared to DMSO controls (177 ± 44 ng/ml; *p* < 0.05) (Fig. 5c). However, BADGE-treated C3FeB6-A/A^w-j^*wt/wt* mice reflected similar levels of Lcn2 to DMSO controls (206 ± 21 vs. 177 ± 44 ng/ml, respectively).

## Discussion

This is the first report showing subclinical colonic inflammation coexisting with progressive ankylosis in the *ank/ank* mouse model, which enables the use of *ank/ank* mouse to unravel the mechanisms underlying the gut-joint manifestations. The gut phenotype appeared to be independent of the presence or absence of a functional ank protein, as no distinguishable inflammatory features were found when comparing *ank/ank* and age-matched C3FeB6-A/A^w-j^*wt/wt* mice, except for higher degree of mucin depletion in the *ank/ank* mice. However, the contribution of *ank* gene to the concurrent gut-joint pathogenesis in *ank/ank* mutant mice was not the main focus of this study. These animals provided us the opportunity to interpret the gut-joint overlap in the context of aberrant pathways in coexisting gut-joint abnormality.

In the *ank/ank* mouse model, the discovery of elevated Lcn2 associated with subclinical colonic inflammation and ankylosis is a novel serological feature linking the gut-joint entities. The occurrence of ankylosis and severe subclinical gut inflammation with increasing serum Lcn2 levels implicates a possible synergistic contribution of gut and bone to circulating Lcn2 levels since neither gut inflammation alone in C3FeB6-A/A^w-j^*wt/wt* mice nor ankylosis alone in *ank/ank* mutants was reflected in increased serum levels of Lcn2. Interestingly, in the group of *ank/ank* mice with fully fused spine but minimal gut inflammation (pathology score 0), various levels of Lcn2 were detected. A possible explanation is the various host-microbe interactions (not reflected in the degree of gut inflammation) in *ank/ank* mice. Since Lcn2 is an anti-microbial factor as reflected in its iron binding function during acute infection [33, 34], levels of Lcn2 could potentially reflect changes of microbiome in the gut. Thus, the increase of Lcn2 in *ank/ank* mutants with severe gut inflammation (pathology score 5 or 6) could be attributed to not only inflammation, but also host-microbes interactions of this strain. This implicates a potential involvement of gut microbiome in the gut-joint axis of diseases. While increased Lcn2 could play a role in the subsequent inflammatory response [35], the downstream inflammatory cytokines (such as IL-17 and TNFα) would enhance Lcn2 expression by a feedforward loop [36]. Thus, it is conceivable that the secondary upregulation of Lcn2 may persist by the ongoing inflammation, which in turn would have an effect on chronic gut inflammation as well as bone homeostasis [13, 34, 37–39].

Outstanding issues in this study which require clarifications include (1) a direct association of Lcn2 levels and the changes of gut microbiome. (2) As activated cells can produce more factors even though the total cell numbers might be the same, the proportion of activated infiltrates in *ank/ank* vs. C3FeB6-A/A^w-j^*wt/wt* mice remains to be investigated. (3) Whether the lack of significant association of serum Lcn2 with pathology scores in the C3FeB6-A/A^w-j^*wt/wt* mice is due to under-powering requires confirmation. (4) In this study, *ank/ank* mice were aged to 4–5 months, at which time all displayed full-blown ankylosis to control the phenotypic variation of ankylosis during aging. It is not known whether the extent of histological difference in the axial and peripheral joints of the mutants differs at the most advanced stage of full-blown ankylosis.

The upregulation of LCN2 in human patients with comorbidities of AS and IBD confirmed the findings in *ank/ank* mice, implicating that both gut and joints are among the sources of circulating lipocalin 2. This is further demonstrated by comparing AS patients (with or without IBD) with different degrees of spinal ankylosis. At present, it remains unclear what tissues are the primary producers of elevated systemic LCN2 levels in humans. In mice, most systemic Lcn2 is derived from hepatocytes [40, 41]. It is likely that resident cells (e.g., IECs [14], Paneth cells [14], and osteoblasts [15]) and immune cells (e.g., macrophages [42] and neutrophils [43]) are responsible for the local upregulation of Lcn2. It remains unresolved regarding the conditions required for local increase of Lcn2 to be sufficient leading to systemic upregulation. Similar to *ank/ank* mice with minimal subclinical gut inflammation, it is of note that some patients with AS alone had elevated LCN2 levels, compared to IBD patients and healthy controls. In addition to various host-microbe interactions, another possible explanation is the lack of information regarding subclinical gut inflammation of these patients. The current protocol did not include colonoscopy screening. It has been reported that in AS patients, elevated serum calprotectin and CRP are independently associated with subclinical gut inflammation [43]. The unknown subclinical articular features in IBD only patients may contribute to the 31% of these patients who had elevated LCN2 levels. There is a recent publication reporting normal LCN2 levels in 21 patients with full-blown AS [44]. The contradictory result is likely due to small sample size and unknown clinical or subclinical gastrointestinal features in this published report.

The modulation of Lcn2 by the PPARγ agonist Rosi in the C3FeB6-A/A^w-j^*wt/wt* mice indicates a potential involvement of PPARγ in the aberrant Lcn2 pathway in the gut-joint axis. It is known that Rosi modulates NFκB activation through nuclear translocation of PPARγ [45]. As there is a binding site for NFκB within the promoter region of gene coding Lcn2 [46], the treatment with Rosi would likely activate the expression Lcn2 through PPARγ/NFκB pathways, which yet remains to be confirmed by immunohistochemistry studies. This finding is consistent with an *S*. *typhimurium*-induced colitis model, where the absence of PPARγ in intestinal epithelial cells was accompanied by lower colonic Lcn2 expression after infection [17]. The reason why Rosi effect was not detected in *ank/ank* mice is likely attributed to high (saturated) baseline Lcn2 levels in these animals compared to C3FeB6-A/A^w-j^*wt/wt* controls. The lack of significant changes after BADGE treatments could be due to the short treatment window of the study design, which is consistent with two experimental-induced colitis rat models both treated with BADGE [47, 48]. Compared to Rosi treatment results, the contradictory inverse association of Lcn2 and PPARγ at baseline is possibly due to the different source of serum Lcn2 vs. colonic PPARγ. A specific association of Lcn2 and PPARγ at the same effector sites (gut vs joint) has yet to be established. It is unclear whether the circulating Lcn2 levels reflect local expression of Lcn2. As mentioned earlier, most circulating murine Lcn2 is derived from hepatocytes [40, 41], while the local upregulation of Lcn2 relies on resident cells [14, 15] and immune cells [42, 43]. This may explain the weak association of serum Lcn2 and colonic PPARγ at baseline. Contradictory results on the association of PPARγ and Lcn2 in different organs have been reported [14, 22]. The reason(s) for discrepancies is unclear. In our study, despite PPARγ being shown to influence the levels of circulating Lcn2, the role of PPARγ in modulating gut and joint phenotypes via the regulation of Lcn2 remains to be assessed. A mixed group of *ank/ank* mice with various degrees of colon inflammation were used, and there is no association between fecal Lcn2 and the severity of colon inflammation. This is likely due to low number of neutrophil infiltrates in these mice, since LCN2 is a unique marker of neutrophil inflammation in patients with ulcerative colitis [49]. It remains unclear whether the most commonly used fecal neutrophil-produced calprotectin would be informative in our mice [50]. Rather than colonoscopy in live animals, a non-invasive method of analyzing colon inflammation before treatment has yet to be validated. The relationship of LCN2 and PPARγ in human patients remains to be established as well.

To clarify a direct contribution of Lcn2 in the pathogenesis of gut-joint manifestations, further mechanistic studies could be helpful. The link between PPARγ and Lcn2 could be demonstrated by analyzing the expression of functional genes in Rosi/BADGE-treated intestinal epithelial cell lines (e.g., HT-29, HCT116, DLD-1, T84 [16]) and bone cell lines (e.g., MC3T3-E1, ST2 [51]). Manipulating Lcn2 expression in *ank/ank* mice (e.g., cross-breeding with Lcn2 deficient mice) may also provide additional information. There are limitations in the use of the *ank/ank* mouse model, even though Lcn2 is intrinsically increased in these mice. As studies on bone homeostasis require a long-term design, blockage of Lcn2 or administration of recombinant Lcn2 might be limited by the short lifespan of less than 6 months of these animals. Both Lcn2^Hep−/−^ and global Lcn2^−/−^ mice were demonstrated to have an increased susceptibility to bacterial infection [41, 52]. Suppressing the expression of Lcn2 followed by a bacterial infection may be harmful or lethal in *ank/ank* mice which already have subclinical gut inflammation. A better animal model with gut-joint manifestations and association with Lcn2 has yet to be identified to resolve these issues.

In summary, the dysregulation of lipocalin 2 associated with coexisting gut inflammation and ankylosis in the *ank/ank* mouse model and human patients indicates a novel mechanism for inflammation and ankylosis of gut-joint coexistence. As elevated LCN2 levels implicate ongoing gut inflammation and progressive spinal ankylosis, our findings suggest that normalization of LCN2 through modulation of PPARγ could be viewed as a treatment target in AS and IBD. Further long-term validation studies are required before this concept can be translated into modification of treatment recommendations.

## Conclusions

The association of lipocalin 2 with concurrent inflammation and ankylosis in the *ank/ank* mice and human patients indicates that lipocalin 2 could be a potential pathway involved in AS and IBD. Therapies regulating PPARγ could be novel treatments for AS targeting the lipocalin 2 pathway.

## Supplementary information

**Additional file 1: Figure S1.** Neutrophil and plasma cell counts in mice. Neutrophil count (A) and plasma cell count (B) were compared between *ank/ank* mice (*n* = 40) versus C3FeB6-A/A^w-j^*wt/wt* mice (*n* = 21). Student’s t-test was used. **Figure S2** Correlation of the primary and secondary pathology scoring systems. A. Correlation of primary and secondary pathology scores. B. Correlation of primary pathology scores and neutrophils counts. C. Correlation with primary pathology scores and plasma cell counts. Pearson’s correlation coefficient test was used. **Figure S3** Correlation of fecal Lcn2 levels and colon pathology in mice. A. Fecal levels of Lcn2 in *ank/ank* mice and C3FeB6-A/A^w-j^*wt/wt* mice with different pathology scores. B. Fecal levels of Lcn2 vs. total protein in *ank/ank* mice and C3FeB6-A/A^w-j^*wt/wt* mice with different pathology scores. Pearson’s correlation coefficient test was used. **Figure S4** Correlation of serum LCN2 levels and mSASSS in patients with AS only. Detection of serum LCN2 levels of different degrees of ankylosis (mSASSS) in AS patients. Pearson’s correlation coefficient test was used. **Table S1.** Comparison of the severity of subclinical inflammation in *ank/ank* vs. C3FeB6-A/A^w-j^*wt/wt* mice.

## Data Availability

Data sharing is not applicable to this article as no datasets were generated or analyzed during the current study.

## Abbreviations

AS: Ankylosing spondylitis
BADGE: Bisphenol A diglycidyl ether
IBD: Inflammatory bowel diseases
Lcn2/LCN2: Lipocalin 2
MBP: Mechanical back pain
mSASSS: Modified Stoke AS Scoring System
PPARγ: Nuclear receptor peroxisome proliferator-activated receptor gamma
Rosi: Rosiglitazone
