# Supplementary material for: Lipocalin 2 links inflammation and ankylosis in the clinical overlap of inflammatory bowel disease (IBD) and ankylosing spondylitis (AS)
Source: Arthritis Res Ther. 2020 Mar 18;22:51. doi: 10.1186/s13075-020-02149-4 (PMC7081573; doi:10.1186/s13075-020-02149-4)
Supplement: Supplementary file 1 — Additional file 1: Figure S1. Neutrophil and plasma cell counts in mice. Neutrophil count (A) and plasma cell count (B) were compared between ank/ank mice (n = 40) versus C3FeB6-A/Aw-jwt/wt mice (n = 21). Student’s t-test was used. Figure S2 Correlation of the primary and secondary pathology scoring systems. A. Correlation of primary and secondary pathology scores. B. Correlation of primary pathology scores and neutrophils counts. C. Correlation with primary pathology scores and plasma cell counts. Pearson’s correlation coefficient test was used. Figure S3 Correlation of fecal Lcn2 levels and colon pathology in mice. A. Fecal levels of Lcn2 in ank/ank mice and C3FeB6-A/Aw-jwt/wt mice with different pathology scores. B. Fecal levels of Lcn2 vs. total protein in ank/ank mice and C3FeB6-A/Aw-jwt/wt mice with different pathology scores. Pearson’s correlation coefficient test was used. Figure S4 Correlation of serum LCN2 levels and mSASSS in patients with AS only. Detection of serum LCN2 levels of different degrees of ankylosis (mSASSS) in AS patients. Pearson’s correlation coefficient test was used. Table S1. Comparison of the severity of subclinical inflammation in ank/ank vs. C3FeB6-A/Aw-jwt/wt mice. [file 13075_2020_2149_MOESM1_ESM.pdf]

Supplementary Figure 1 Neutrophil and plasma cell counts in mice. Neutrophil count (A) and plasma cell count (B) were compared between *ank/ank* mice (n=40) versus C3FeB6-A/Aw-j *wt/wt* mice (n=21). Student's t-test was used.

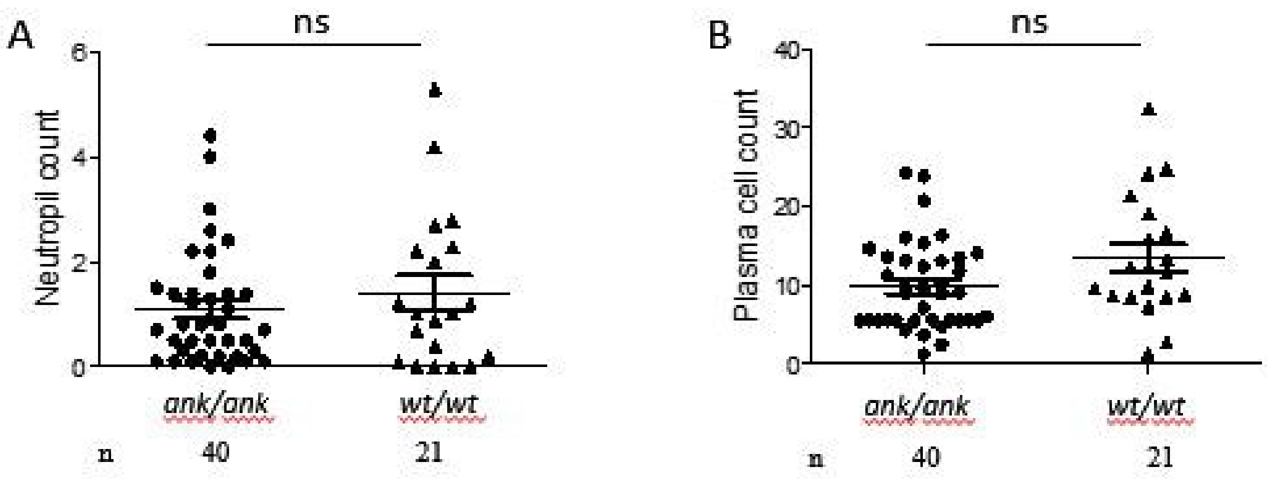

Supplementary Figure 2 Correlation of the primary and secondary pathology scoring systems. A. Correlation of primary and secondary pathology scores. B. Correlation of primary pathology scores and neutrophils counts. C. Correlation with primary pathology scores and plasma cell counts. Pearson's correlation coefficient test was used.

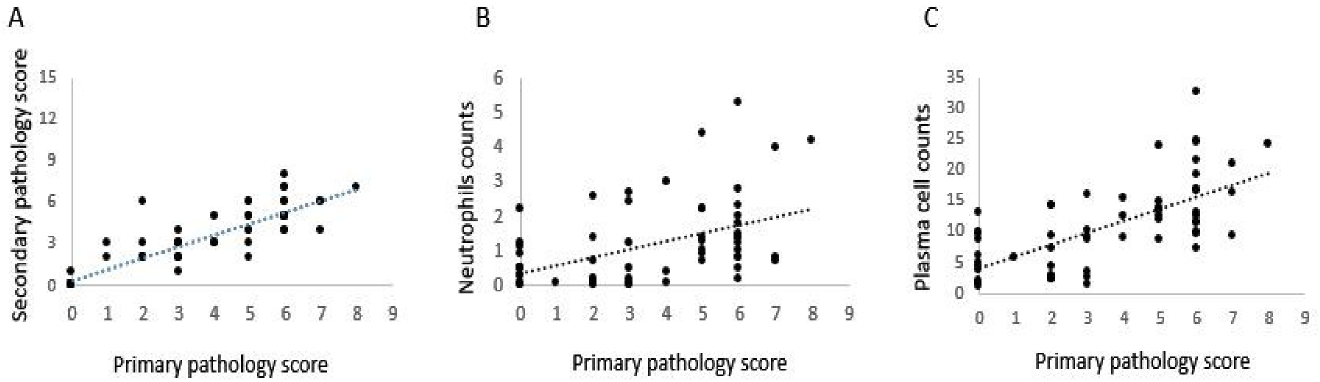

Supplementary Figure 3 Correlation of fecal Lcn2 levels and colon pathology in mice. A. Fecal levels of Lcn2 in *ank/ank* mice and C3FeB6-A/Aw-j *wt/wt* mice with different pathology scores. B. Fecal levels of Lcn2 vs. total protein in *ank/ank* mice and C3FeB6-A/Aw-j *wt/wt* mice with different pathology scores. Pearson's correlation coefficient test was used.

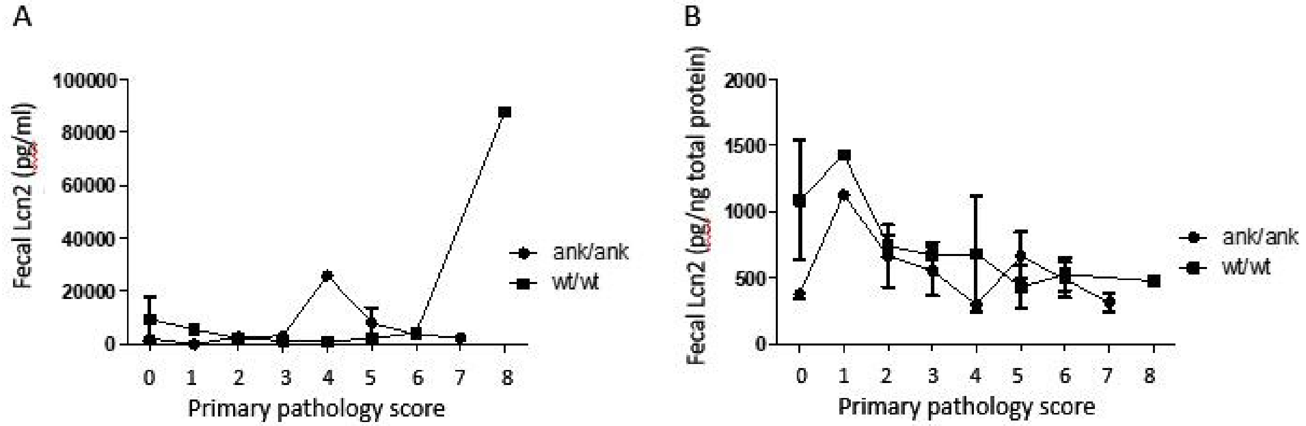

Supplementary Figure 4 Correlation of serum LCN2 levels and mSASSS in patients with AS only. Detection of serum LCN2 levels of different degrees of ankylosis (mSASSS) in AS patients. Pearson's correlation coefficient test was used.

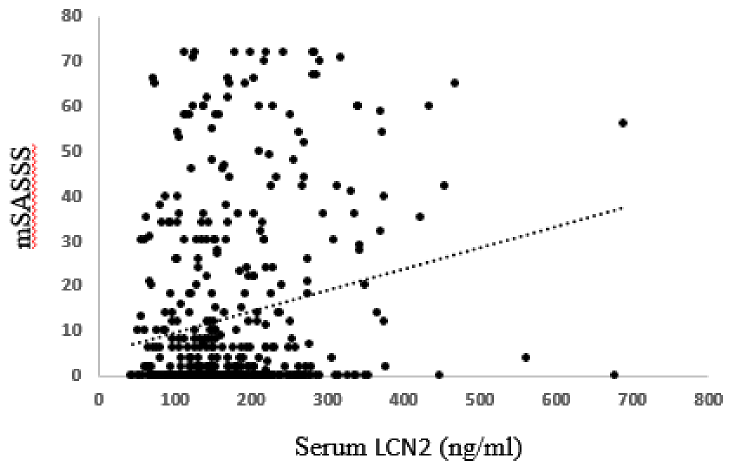

Supplementary Table 1 Comparison of the severity of subclinical inflammation in *ank/ank* vs. C3FeB6-A/Aw-j *wt/wt* mice.

Genotype	n	Mucin Depletion (0-3)	Hyperplasia (0-3)	Number of foci (0-3)	Architectural distortion (0-3)	Granuloma (0-3)	Depth of inflammation (0-3)
ank/ank	40	0.03±0.03	0.8±0.2	1.0±0.1	0.03±0.03	0	1.0±0.1
C3FeB6-A/Aw-j *wt/wt*	21	0.3±0.1 **	1.1±0.2	1.3±0.2	0	0	1.2±0.2
